# M. M. Smith, M. D., Dallas, Texas

**Published:** 1918-11

**Authors:** 


### M. M. Smith, M. D., Dallas, Texas.

---

There are men here and there in the medical profession of whom we feel proud, and whom we delight to present to our readers as examples of the best ideals of a noble calling. No man is more worthy of this honor than that well-poised soul of honor, sentiment, and sincerity, Dr. M. M. Smith of the State of Texas.

A man of splendid physique, delightful personality and magnetism, high principles and strong character, he has stood out in bold relief for more than a quarter of a century as a leader in the profession in his State for all that goes to elevate and purify the standard of professional excellence and ethics. Dr. Smith is a cultured man, having received the degrees of B. Sc., M. A. and M. D., the latter from honorable old Jefferson, of Philadelphia, in 1891.

He practiced medicine for many years in Austin, Texas; was physician to the Texas Deaf and Dumb Institute for twelve years, at Austin, resident physician for four years at the city and county hospital at Austin, was a member and Secretary of the Board of Medical Examiners for Texas and has always been active in medical legislation, serving each year, for some ten or fifteen years, on the Legislative Committee, which procured for Texas, laws regulating the practice of medicine within the State, the establishment of the State Board of Health and institutions for the care of the Tuberculous, for Texas. He was Secretary of the International Tuberculosis Congress, which held its meeting in New York City in 1901, and President of the Medical Section of the National Fraternal Congress of America which had its meeting in Cleveland, O., in 1916, and is Medical Director of the Modern Order of Praetorians—a life insurance company—and is still actively holding this position.

With all his honors and successes, and the many real accomplishments he has wrought for the profession and humanity, he is yet in his prime, a bright, genial, broad and lovable soul carrying sunshine wherever he goes—a man of genuine parts—a friend as staunch as steel.—The Southern Clinic.

---

True Pleasure.—"Major Rasher, I saw a man today who would like the pleasure of kicking you," said a friend.

"Kicking me!" exploded the Major. "Kicking me! Give me his name at once!"

"I hardly like to tell you," said the other.

"I insist upon knowing," said the Major.

"Ah, well, I'll tell you," said the other. "It's a soldier who's in the hospital with both legs off."—*Tit-Bits.*

